# The Difference a Year Can Make: How Antibiotic Resistance Mechanisms in *Pseudomonas aeruginosa* Have Changed in Northwestern Transylvania

**DOI:** 10.3390/biom15010001

**Published:** 2024-12-24

**Authors:** Matei-Ștefan Dobrescu, Dan-Alexandru Țoc, Adrian-Gabriel Pană, Carmen Costache, Anca Butiuc-Keul

**Affiliations:** 1 Doctoral School of Integrative Biology, Babeș-Bolyai University, 1 M. Kogalniceanu Street, 400084 Cluj-Napoca, Romania; matei.dobrescu@ubbcluj.ro; 2 Department of Molecular Biology and Biotechnology, Faculty of Biology and Geology, Babeș-Bolyai University, 1 M. Kogalniceanu Street, 400084 Cluj-Napoca, Romania; 3 Centre for Systems Biology, Biodiversity and Bioresources, Babes-Bolyai University, 5–7 Clinicilor Street, 400006 Cluj-Napoca, Romania; 4 Department of Microbiology, Iuliu Hatieganu University of Medicine and Pharmacy, 400012 Cluj-Napoca, Romania; 5 Cluj County Emergency Hospital, 400000 Cluj-Napoca, Romania; 6 Faculty of Medicine, Iuliu Hatieganu University of Medicine and Pharmacy, 400012 Cluj-Napoca, Romania

**Keywords:** antibiotic resistance genes, antibiotic resistance mechanisms, integrons, multidrug-resistant bacteria, *Pseudomonas aeruginosa*

## Abstract

This study examines the prevalence and the mechanisms of antibiotic resistance in *Pseudomonas aeruginosa* isolates collected from healthcare units in Northwestern Transylvania, Romania, between 2022 and 2023. Given the alarming rise in antibiotic resistance, the study screened 34 isolates for resistance to 10 antibiotics, 46 ARGs, and integrase genes using PCR analysis. The results reveal a concerning increase in multidrug-resistant (MDR) and extensively drug-resistant (XDR) isolates over the two-year period. Notably, the prevalence of ARGs encoding resistance to sulfonamides and beta-lactams, particularly *sul1* and *bla_OXA-50_*, has shown a significant rise. Furthermore, the study detected the emergence of new resistance mechanisms in the same time interval. These include target protection and even more specific mechanisms, such as metallo-beta-lactamases or enzymes involved in the methylation of 23S rRNA. Statistical analysis further confirmed the correlation between Class I integrons and several ARGs, underscoring the role of horizontal gene transfer in the dissemination of resistance. These findings emphasize the urgent need for updated treatment strategies and monitoring programs to effectively combat the spread of ARGs in clinical settings.

## 1. Introduction

Antibiotics play a crucial role in the treatment and prevention of diseases caused by harmful bacteria, dramatically lowering mortality rates [1]. The 20th century was widely regarded as the ‘golden age’ of antibiotics, during which they were extensively used in both humans and animals. Their widespread use significantly reduced the incidence and severity of infectious diseases globally, leading to a dramatic decrease in morbidity and mortality. Notably, antibiotic consumption has continued to rise in the 21st century, likely due to the improved access to these medications in low- and middle-income countries. In fact, global antibiotic consumption increased by a substantial 65% between 2000 and 2015, coinciding with the remarkable decline in deaths caused by infectious diseases [2].

Antimicrobial resistance poses an alarming global hazard to human health, defined by bacteria’s acquisition of resistance to the bacteriostatic or bactericidal effects of antibiotics, complicating the treatment of diseases caused by these pathogens [1]. Over the past ten years, antibiotic-resistant bacteria (ARB) and antibiotic resistance genes (ARGs) have been found in various types of habitats, including natural and clinical ones. Most scientists agree that anthropogenic activities, such as the use of antibiotics in clinical settings, are the primary factors contributing to the increased resistance in bacteria and ARGs [3].

Horizontal gene transfer (HGT) significantly contributes to bacterial diversity by facilitating the exchange of genetic material among unrelated bacterial cells, enabling microorganisms to acquire traits that enhance their adaptation to novel or stressful environments, optimize resource utilization, and defend against harmful substances [4]. Horizontal gene transfer significantly contributes to the medical crisis posed by antibiotic-resistant pathogenic bacteria, whose number is rising due to the acquisition of ARGs via mechanisms such as conjugation or through mobile genetic elements like integrons [1]. Integrons have a significant role in dissemination during antibiotic treatment, being capable of re-arrangements through a “copy–paste” model, thus creating copies of a gene, increasing resistance to a certain antibiotic. This was identified for ARGs such as *ant(2″)-Ia* (*aadB*). Another mechanism is cassette duplication in relevant plasmids, observed in plasmids carrying the *bla_VIM-1_* gene [5].

The World Health Organization identifies 12 pathogenic bacteria responsible for nosocomial infections, with *Pseudomonas aeruginosa* categorized in the highest risk class, prioritizing the development of novel treatments [6]. *Pseudomonas aeruginosa* is a Gram-negative bacterium, round-cylindrical, and facultatively anaerobic; the structures involved in anaerobic survival help the species persist and develop in unfavorable environments [7]. These bacteria, which are found in the environment and are a part of the gut flora, can lead to respiratory and urinary tract infections, particularly in those with impaired immune systems [8].

Bacteria focused on becoming equipped to avoid, dispose of, or adapt to the substances that endangered them as they evolved [7]. The bacterial genome has undergone a number of mutations over time that have caused the overexpression of genes, such as those encoding enzymes that render antibiotics inactive or genes encoding efflux pumps. This has led to the bacteria becoming resistant to a wide range of antibiotic classes [9]. Acquiring resistance to at least three classes of antibiotics will render the bacteria multidrug-resistant (MDR) [10], while others can become extensively drug-resistant (XDR), being resistant to all classes of antibiotics, except one or two [11].

Most studies focus on the phenotypic identification of antibiotic resistance, which does not necessarily show a complete image of the resistance profile of an isolate/strain [12,13,14]. In Romania, the situation is even worse; only a few studies focus on the genotypic investigation of several pathogens usually found in clinical settings or environmental samples. Unfortunately, there are no studies regarding the comprehensive analysis of the phenotypic and genotypic resistance of the most important bacterial pathogens involved in nosocomial infections, across the years, making it difficult to observe and follow the evolution and spread of antibiotic resistance genes. Therefore, this study aims to investigate the pattern of resistance and the prevalence of several ARGs in *P. aeruginosa* isolates from healthcare units from NW Transylvania, Romania, during the COVID post-pandemic (2022–2023), focusing on the novel mechanisms of resistance acquired by bacteria because of selective pressure of antibiotics used in therapy.

## 2. Materials and Methods

### 2.1. Materials

Bacterial isolates of *P. aeruginosa* have been collected between January 2022 and December 2023 (22 in 2022 and 12 in 2023) from 2 hospitals from Northwestern Transylvania (Cluj Emergency County Hospital and Satu Mare Emergency County Hospital). Species identification was conducted using the VITEK-2 Compact system, specifically the VITEK^®^ 2 GP ID card (bioMérieux, Inc., Marcy l’Etoile, France), and phenotypic resistance profiles to 10 antibiotics were determined using the VITEK^®^ 2 AST-P592 card (bioMérieux, Inc., Marcy l’Etoile, France). All isolates were obtained under full anonymity and ethics agreements were signed.

All isolates were screened for 46 ARGs, as well as integrase genes for class I, II, and III integrons by PCR amplification. Specific primers (Eurogentec, Belgium) were used to amplify genes encoding antimicrobial resistance and integron integrase (as listed in Supplementary Table S1).

### 2.2. Methods

**Figure 1:**
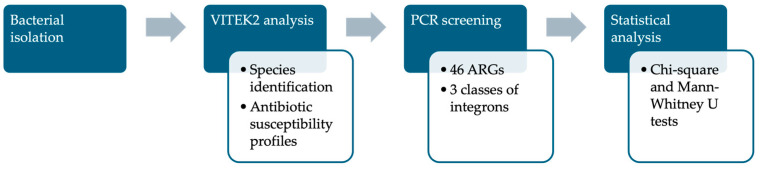
Workflow of the analysis of the antibiotic resistance of *P. aeruginosa* isolates.

PCR reactions were conducted in a total volume of 25 μL, comprising 12.5 μL of DreamTaq Green PCR master mix (2×) (Thermo Fisher Scientific, Whaltman, MA, USA), 10.25 μL of nuclease-free water (Lonza, Basel, Switzerland), 25 pmol of each primer (Eurogentec, Liège, Belgium), and 2 μL of bacterial suspension. DNA isolation was omitted, and the PCR technique utilized bacterial suspensions as templates. *Pseudomonas aeruginosa* cultures were diluted in sterile water to achieve a concentration of approximately 10^6^ cells/mL [15]. The PCR reaction was mixed with 2 μL of sterile water as a negative control. The standard PCR protocol was as follows: 4 min at 94 °C, followed by 35 cycles of 30 s at 94 °C, 45 s at a certain annealing temperature (based on the primers used, as indicated in Supplementary Table S1), and an 8-minute final extension at 72 °C. PCRs were performed using a thermocycler TProfessional Trio (Analytik Jena, Jena, Germany), Mastercycler Nexus (Eppendorf AG, Hamburg, Germany), or Gradient Palm-Cycler (Corbett Life Science, Sydney, Australia). The amplicons were separated in 1.5% *w*/*v* agarose gel (Cleaver Scientific, Rugby, Warwickshire, UK) in 1 × TAE buffer (Lonza, Switzerland), and then stained with 0.5 μg/mL ethidium bromide (Thermo Fisher Scientific, USA). BioDocAnalyze software (Analytik Jena, Germany) and the BDA Digital Compact System were used to acquire the data. A workflow of the analysis of the phenotypic and genotypic antibiotic resistance of *P. aeruginosa* is presented in Figure 1.

### 2.3. Data Analysis

Statistical tests were made by using the R (ver. 4.4.1, The R Foundation, Vienna, Austria) and RStudio software (ver. 2024.09.0+375, Posit Software, Boston, MA, USA). The applied tests were Mann–Whitney U for correlations and Chi-test for significance [16], all available in R packages. For all tests, a *p* value under 0.05 is considered significant.

## 3. Results

The isolates of *P. aeruginosa* were obtained from samples originating from urine, purulent collections, tracheal aspirates, blood, plague secretions, and pleural or pericardial fluids. The VITEK2 screening identified the resistance and susceptibility to 10 antibiotics (amikacin, cefepime, ceftazidime, ciprofloxacin, colistin, imipenem, meropenem, piperacillin-tazobactam, piperacillin, and tobramycin). Most isolates from 2023 are resistant to nine antibiotics and susceptible to one, colistin. In contrast, samples from 2022 have a wider range of susceptibility profiles, half of the samples being resistant to none or at most one antibiotic, while the rest are resistant to almost all screened antibiotics. Isolates obtained from urine samples show a broader resistance pattern, while isolates from tracheal aspirates are susceptible to all screened antibiotics. A small number of isolates (4 out of 34) are resistant to colistin, while 10 out of the 34 screened isolates were identified to be susceptible to all antibiotics used in the VITEK 2 screening (Supplementary Table S2). All isolates were characterized as MDR when tested by VITEK 2.

Out of all isolates, 70.59% are resistant to piperacillin, 70.59% to ciprofloxacin, and 67.65% to ceftazidime. In opposition, 58.82% of the isolates are resistant to amikacin, 55.88% to piperacillin-tazobactam, and only 11.76% are resistant to colistin. Comparing 2022 to 2023, the prevalence of the isolates resistant to the antibiotics tested has increased, mostly for ciprofloxacin (from 54.55% to 100%), piperacillin (from 54.55% to 100%), and cefepime (from 54.55% to 91.67%), while the lowest increases were for piperacillin-tazobactam (from 50% to 66.67%), meropenem (from 54.55% to 66.67%), and amikacin (from 54.55% to 66.67%). Out of all these, only the increases for ciprofloxacin and piperacillin resistance are significant (Chi-test, *p* < 0.05).

**Figure 2:**
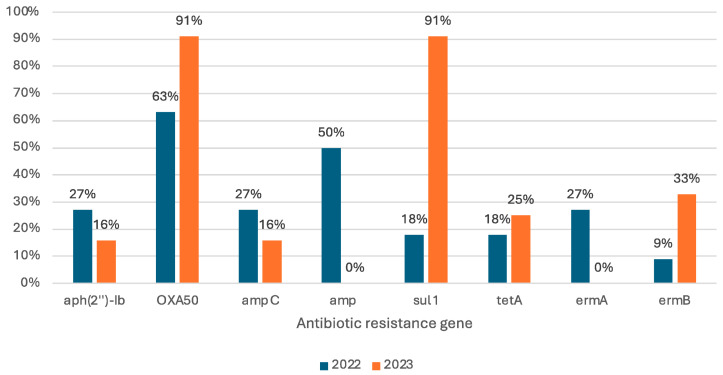
Prevalence of commonly identified ARGs in *P. aeruginosa* isolates between 2022 and 2023.

Out of the 46 screened ARGs, we identified 26 resistance genes which encode the resistance to the following classes of antibiotics: beta-lactams, aminoglycosides, sulfonamides, tetracyclines, macrolides, quinolones, nitrofurans, and phosphonic acid. An increased prevalence can be observed from 2022 to 2023 for the *bla_OXA-50_* gene from 63% to 91% and for the *sul1* gene from 18% to 91%. However, in 2023, several ARGs such as *amp* and *ermA* were not found in the analyzed isolates and the prevalence of *aph(2″)-Ib* and *ampC* genes decreased. Out of all these genes, we only found a statistically significant increase in the prevalence of the *sul1* gene and a statistically significant decrease in the prevalence of the *amp*C gene (Chi-test, *p* < 0.05) (Figure 2). The most prevalent ARGs are *bla_OXA-50_* (25 out of 34 isolates), *sul1* (18 out of 34 isolates), and *amp* (11 out of 34 isolates).

**Figure 3:**
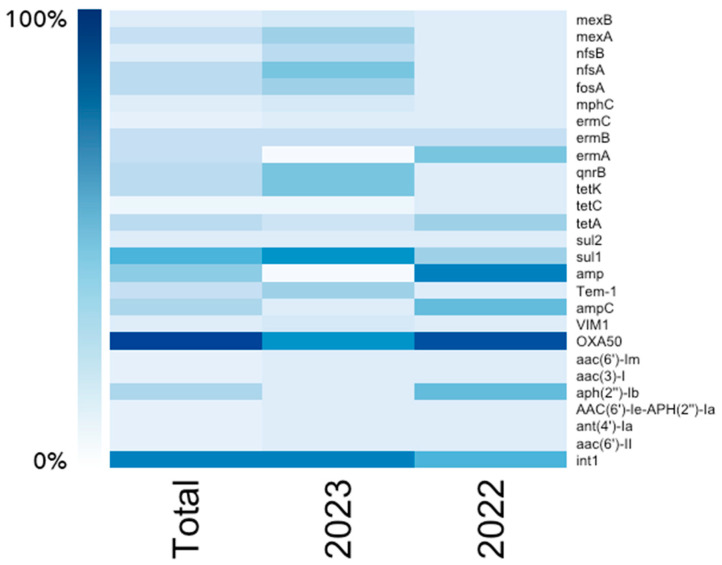
Prevalence of ARGs in P. aeruginosa isolates by year.

The following ARGs were identified in 2022: *aph2″-Ib*, *bla_OXA-50_*, *ampC*, *amp*, *sul1*, *tetA*, *ermA*, and *ermB*. In 2023, over 20 ARGs have been identified, such as *fosA*, *nfsA*, *nfsB*, *mexA*, and *mexB*, which relate to resistance to phosphonic acid, nitrofurans, and broad-spectrum resistance (Figure 3). In both 2022 and 2023, *bla_OXA-50_* has a high prevalence, followed by *sul1*. In 2023, more ARGs encoding resistance to beta-lactams (*bla_VIM-1_* and *TEM-1*), sulfonamides (*sul2*), tetracyclines (*tetC* and *tetK*), macrolides (*ermC* and *mphC*), and aminoglycosides (*aac(6′)-II*, *ant(4′)-Ia*, *aac(6′)-Ie-aph(2′)-Ia*, *aac(3)-I*, and *aac(6′)-Im*) were identified.

Regarding the mechanisms of resistance, most identified ARGs encode proteins that inactivate an antibiotic, by destroying the beta-lactam ring or by modifying the antibiotic molecule. In 2022, only ARGs encoding the enzymes phosphotransferase and serine beta-lactamase were identified, while, in 2023, new genes were found encoding 6′-N-acetyltransferase, acetyltransferase, and metallo-beta-lactamase. In 2023, we identified the *ermC* gene in the analyzed isolates which encodes an enzyme involved in the methylation of 23S rRNA in order to modify the antibiotic target, with the enzyme not being present in 2022. Compared to 2022, in 2023, the emergence of a new resistance mechanism such as target protection, encoded by genes like *qnrB* (Table 1), was observed.

**Figure 4:**
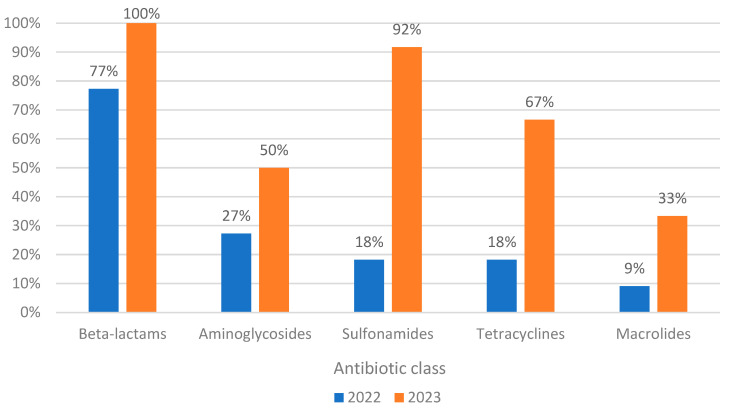
Prevalence of genotypic resistance to commonly identified classes of antibiotics in *P. aeruginosa* isolates between 2022 and 2023.

In 2023, genotypic resistance increased considerably, such as, for example, in sulfonamides, from 18% to 92%, followed by tetracyclines, from 18% to 67%. Both increases are statistically significant (Chi-test, *p* < 0.05). In 2023, all screened isolates were resistant to beta-lactams. Compared to 2022, isolates from 2023 are also resistant to quinolones, nitrofurans, and phosphonic acid (Figure 4).

**Figure 5:**
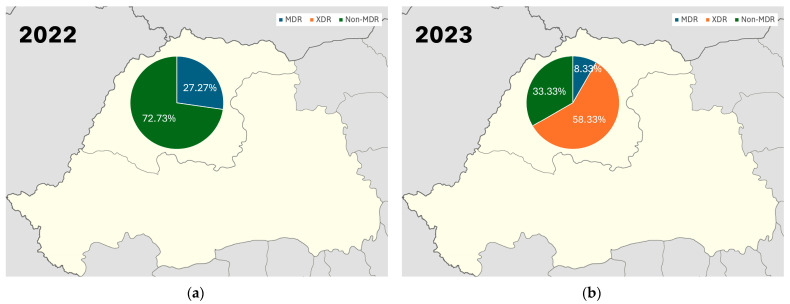
Prevalence of MDR *P. aeruginosa* isolates between 2022 (**a**) and 2023 (**b**).

Out of 34 isolates, 20% are MDR (7 isolates), 21% are XDR (7 isolates), and 59% are non-MDR (20 isolates). In 2022, 72.73% of the isolates were non-MDR and 27.27% were MDR. In 2023, XDR isolates represent 58.33% of the isolates. MDR isolates have decreased to 8.33% and non-MDR isolates represent 33.33% (Figure 5).

**Figure 6:**
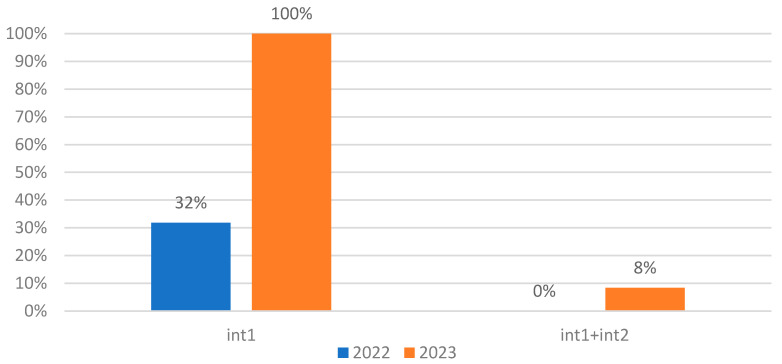
Prevalence of integrons in *P. aeruginosa* isolates between 2022 and 2023.

The class I integron was identified in 54% of isolates and 43% do not contain integrons at all. It is important to note that one isolate (3% of total) contains both class I and II integrons. An increase in the number of isolates bearing the class I integrons between 2022 and 2023 from 32% to 100% has been observed. There are no isolates harboring only class II integrons (Figure 6).

## 4. Discussion

Through the VITEK 2 screening, the resistance to 10 antibiotics such as amikacin, cefepime, ceftazidime, ciprofloxacin, colistin, imipenem, meropenem, piperacil-tazobactam, piperacillin, and tobramycin (corresponding to four classes of antibiotics: aminoglycosides, beta-lactams, polymyxins, and fluoroquinolones) was identified throughout the isolates. According to the phenotypic pattern of resistance, all selected isolates were reported as MDR. However, due to the low number of antibiotics tested (according to the hospital protocol) and the high prevalence of class I integrons in isolates, we assumed that genotypic resistance could be even higher, and a more comprehensive ARG screening was performed. Thus, several ARGs were analyzed for each antibiotic tested, but other ARGs encoding the resistance to sulfonamides, macrolides, and tetracyclines were tested as well, according to Supplementary Table S3. The results regarding the ARG screening confirmed our assumption: 55.88% (19 out of 34) of the isolates showed complex patterns of ARGs, encoding the resistance to many more other classes of antibiotics than phenotypically tested. There are also cases where the phenotypic resistance was not confirmed by the presence of the ARGs tested, such as, for example, P1_22, which is resistant to piperacillin, but the *ampC* gene was not identified, or isolates P3_22 and P4_22 presenting resistances to amikacin, cefepime, ceftazidime, ciprofloxacin, imipenem, meropenem, piperacillin, and tobramycin, but the associated ARGs were not identified by PCR screening. The possible explanations could be that the resistance to those antibiotics is encoded by other genes which were not investigated in this study or due to the putative mutations in the ARG sequences that do not allow the proper alignment of primers during PCR.

Nevertheless, our results were similar to another study from Spain, in which 243 isolates of *P. aeruginosa* from blood samples were tested for the same antibiotics as in our study, except for piperacillin, but also for gentamycin, ceftolozane-tazobactam, and ceftazidime [18]. When looking at the commonly tested antibiotics in isolates originating from blood, a decrease in resistance for all antibiotics, except colistin, was observed. All these differences are statistically confirmed to be relevant (Chi-square test, *p* < 0.05). This only applies to isolates obtained from blood samples. Another study from the USA, investigating the resistance of 886 MDR isolates of *P. aeruginosa* from multiple sample origins, to amikacin, cefepime, ceftazidime, ciprofloxacin, colistin, imipenem, meropenem, piperacillin-tazobactam, imipenem/relebactam, ceftolozane/tazobactam, aztreonam, and levofloxacin, showed similar results with ours, regarding the resistance to amikacin, cefepime, ceftazidime, ciprofloxacin, colistin, imipenem, meropenem, and piperacillin-tazobactam [19]. For most antibiotics, we did not identify significant changes in resistance; however, we identified a significant increase in colistin and amikacin resistance and a significant decrease in piperacillin-tazobactam resistance.

One of the major shortcomings of the studies regarding the analysis of the antimicrobial resistance of bacteria from clinical settings is that screening of the phenotypic resistance widely varies from study to study, and, consequently, the results are different and difficult to compare between distant regions of the globe, highlighting the need for the standardization of antimicrobial resistance testing in order to ensure consistent and accurate results. Considering that phenotypic testing is performed for antibiotics that are commonly used in therapy in different regions, and due to the high spread of ARGs by the horizontal transfer of genes, especially those placed on mobile genetic elements (MGEs), genotypic testing should include genes encoding resistance to different classes of antibiotics.

Our study showed how *P. aeruginosa* adapts to the continuous pressure exerted by antibiotics in the clinical environment. The intrinsic beta-lactam resistance in *P. aeruginosa* is attributed to multiple factors, including the reduced membrane permeability resulting from mutations that cause the vanishing of the OprD, the activity of the efflux pumps MexAB-OprM or MexCD-OprJ, and the capacity of the bacteria to produce chromosomally encoded beta-lactamases *ampC* [20]. We identified some of the genes *mexA* and *mexB* which were only found in isolates collected in 2023. Regarding the *ampC* gene, a decreased prevalence was observed in 2023 compared to 2022.

The development of resistance encoded by *bla_OXA_*, *GES*, *TEM*, *SHV*, *VIM*, *IMP*, or *NDM* genes, as well as mutations that attempt to alter the antibiotic target or overexpress efflux systems are the two main causes of bacterial multidrug resistance [21,22]. Out of all mentioned genes, we only identified three of them, *bla_OXA-50_*, *TEM-1* and *bla_VIM-1_*; *bla_OXA-50_* is the most prevalent in the isolates collected in 2022 and 2023, while *bla_VIM-1_* and *TEM-1* only appear in isolates collected in 2023. Regarding the mechanisms of resistance, we notice that the isolates collected in 2023 have a higher diversity of beta-lactamases.

Aminoglycosides are also used extensively in the treatment of infections with *P. aeruginosa*; thus, the predominant resistance mechanism to these antibiotics is mediated by the enzymatic modification of the antibiotic via aminoglycoside-acetyltransferases (AACs), aminoglycoside-adenyltransferases (AADs), and aminoglycoside-phosphotransferases (APHs). These enzymes can also be placed on MGEs, enhancing the widely spread of *aphA1*, *aadB*, and *aac (6′)-Ib* that were the most prevalent genes in analyzed isolates [23]. Our results showed that, in 2022, only one gene encoding resistance to aminoglycosides was identified, *aph(2″)-Ib*, encoding an aminoglycoside-phosphotransferase, while, in 2023, we identified genes encoding aminoglycoside-acetyltransferases and aminoglycoside-phosphotransferases, as well as aminoglycoside nucleotidyltransferases (*ant(4′)-Ia)*. Thus, in 2022, only genes encoding aminoglycoside-phosphotransferases were identified.

Phosphonic acids, another class of antibiotics, are also used to treat infections with *P. aeruginosa*, in addition to aminoglycosides. Even though this antibiotic is not as widely used anymore, new treatment schemes use it together with β-lactams, aminoglycosides, or colistin [23]. We identified the resistance to phosphonic acid in isolates collected in 2023 mediated by the *fosA* gene, present in 50% of isolates.

Our study showed that significant differences between 2022 and 2023 appear only in the case of genotypic resistance to sulfonamides and tetracyclines. Regarding the genotypic resistance, a significant increase in the prevalence of the *sul1* gene was observed while the *ampC* gene registered a significant decrease in prevalence. Even though there is a significant difference between 2022 and 2023 regarding the resistance to tetracyclines, the prevalence of the *tetA* gene showed no significant difference (Chi-test, *p* < 0.05), meaning that resistance to these antibiotics may be encoded by genes involved in other resistance mechanisms, mainly efflux pumps.

Another objective of our study was studying the changes in the prevalent mechanisms of resistance in *P. aeruginosa* during 2022–2023. In a broader sense, in 2022, all major mechanisms are present such as antibiotic inactivation, efflux pumps, target replacement, and the modification of the target. In 2023, all of the above-mentioned mechanisms are maintained, with the addition of target protection, as a result of the presence of the *qnrB* gene. Enzymes such as 6′-N-acetyltransferase (*aac(6′)-II*), metallo-beta-lactamases (*bla_VIM-1_)*, thioltransferase (*fosA)*, or nitroreductase (*nfsA* and *nfsB*) are newly acquired in 2023, compared to 2022.

The MexAB-OprM efflux pump confers resistance to a wide range of antibiotics: peptides, sulfonamides, tetracyclines, cephalosporins, carbapenems, fluoroquinolones, and macrolides [24]. We have identified the *mexA* and *mexB* genes in isolates collected in 2023, giving them the ability to survive in the presence of antibiotics even when specific ARGs are not present.

Integrons, playing a crucial role in the dissemination of ARGs, are present in 17% of bacterial chromosomes. A previous study revealed that 58.3% of screened *P. aeruginosa* isolates harbor class I integrons, while class II and III integrons were absent [25]. Our findings mirrored these results, as we detected class I integrons in 54% of isolates and class III integrons were not found. Notably, we only identified one isolate harboring class II integrons, and, even then, class II integrons were exclusively found in conjunction with class I integrons.

Integrons are known to be carriers of sulfonamide resistance genes [26]. Thus, we have identified a correlation between the presence of sulfonamide resistance genes and integrons (Mann–Whitney U test, *p* < 0.05). Sabbagh et al. reported that integrons also carry the resistance to aminoglycosides and macrolides [27]. We have found a correlation between the presence of aminoglycoside resistance genes and integrons, but not between the presence of macrolide resistance genes and integrons. We also observed a correlation between integrons and *sul1*, *aph(2″)-Ib*, and *ermB*, with ARGs found in both 2022 and 2023 (Mann–Whitney U test, *p* < 0.05).

As mentioned before, integrons can play an important role in gene dissemination and the evolution of antimicrobial resistance, as shown in the case of plasmids carrying the bla_VIM-1_ gene [5]. We have identified this gene in three isolates from 2023, while it was not identified in 2022. It is possible that the plasmid carrying this gene was acquired by the bacterial population during this period. A previous study showed that integrons can facilitate the movement of genes that usually do not disseminate through integrons, especially in polluted environments, mostly the genes encoding resistance to aminoglycosides and beta-lactams. The study identified 296 ARGs that can benefit from integron activity in polluted environments (antibiotics), and 62 of these ARGs can use integrons to move around regardless of the environments [28]. Taking into account our results, the *aac(6′)-II*, *bla_VIM-1_*, *sul1*, and *sul2* genes might benefit from integron activity, regardless of the usual dissemination mechanism of a certain ARG (e.g., via plasmids). Out of all these genes, we only identified a correlation between the presence of *sul1* and integrons.

The discovery of *bla_VIM-1_* in the isolates from our study highlights integrons’ crucial role in promoting the acquisition and spread of clinically relevant ARGs. Integrons may also stabilize the expression of genes like *bla_VIM-1_* [5]. This aligns with Wei et al.’s findings that sublethal antibiotic exposure enhances the resistance determinants in *E. coli* [29]. The exact mechanism of how *bla_VIM-1_* emerged in *P. aeruginosa* isolates remains unknown, but the integron-mediated dissemination of *bla_VIM-1_* may emerge and amplify under comparable selective pressures. Antibiotic stress may also augment genomic plasticity, contributing to the increased multidrug resistance observed. Thus, integron activity and selective pressure converge to drive the spread of genes like *bla_VIM-1_* in *P. aeruginosa* populations.

A previous study identified the presence of several beta-lactamases in ESAKAPE pathogens, including *bla_OXA48_*, *bla_VIM_*, *bla_IMP_*, *bla_NDM_*, and *bla_KPC_*. Among these ARGs, we only identified *bla_VIM_*, with three isolates harboring this gene, compared to none in the aforementioned study, in the case of *P. aeruginosa* [30]. In that study, the presence of *bla_OXA48_* was also reported, while we did not identify this gene; however, we identified the *bla_OXA50_* gene.

The information about the mechanism of the resistance to antibiotics in pathogenic bacteria is important for the monitoring of the spreading of the resistant bacteria and ARGs for adapting the treatment schemes. This information can be combined with machine learning [31] in order to predict how ARGs mutate and how antibiotic resistance changes. This new finding could contribute to the understanding of the complex phenomenon of bacterial resistance in order to prevent, and conduct proper management of a new health crisis that is already looming over us.

## 5. Conclusions

Across only two years, we identified that the antibiotic resistance of *P. aeruginosa* isolates from NW Transylvania, Romania has changed. Compared to 2022, in 2023, the emergence of XDR isolates and a decrease in MDR isolates were observed. Moreover, the prevalence of ARGs such as *bla_OXA-50_*, *sul1*, and *ermB* in isolates collected in 2023 was increased compared to 2022. The emergence of new ARGs in 2023 (*sul2*, *ant(4′)-Ia*, *VIM-1*, and *mphC*) was also observed, as well as the emergence of the resistance to antibiotic classes not identified in 2022 (quinolones, nitrofurans, and phosphonic acid).

The correlation between the *int1* gene and several ARGs such as *sul1*, *aph(2″)-Ib*, and *ermB* demonstrates the key role of class I integrons in the dissemination of these ARGs by HGT. In 2023, all the isolates of *P. aeruginosa* harbored the *int1* gene.

The newly acquired ARGs ensured the diversification of the resistance mechanisms. Moreover, the acquisition of the *mexA* and *mexB* genes encoding an efflux pump offers resistance to most antibiotic classes.

## Figures and Tables

**Table 1:** Classification of analyzed ARGs in *P. aeruginosa* isolates by antibiotic class, resistance mechanism, and dissemination [17].

ARG	Class	Resistance Mechanism	Detailed Mechanism	Dissemination
*aac(6′)-II* ^3^	Aminoglycoside	Antibiotic inactivation	6′-N-acetyltransferase	
*ant(4′)-Ia* ^3^			nucleotidyltransferase	plasmid
*AAC(6′)-Ie-APH(2″)-Ia* ^3^			acetyltransferase	plasmid, transposon
*aph(2″)-Ib* ^2^			phosphotransferase	chromosome
*aac(3)-I* ^3^			3-N-acetyltransferase	
*aac(6′)-Im* ^3^			acetyltransferase	plasmid
*bla_OXA-50_* ^2^	Beta-lactam		serine beta-lactamase	chromosome
*VIM-1* ^3^			metallo-beta-lactamase	plasmid
*ampC* ^2^			serine beta-lactamase	chromosome
*TEM-1* ^3^			serine beta-lactamase	
*amp* ^1^				
*sul1* ^2^	Sulfonamide	Target replacement	dihydropteroate synthase	integron
*sul2* ^3^				plasmid
*tetA* ^2^	Tetracycline	Efflux pump		
*tetC* ^3^				plasmid
*tetK* ^3^				
*qnrB* ^3^	Quinolone	Target protection		
*ermA* ^1^	Macrolide	Target modification		
*ermB* ^2^				
*ermC* ^3^			methylation of 23S rRNA	
*mphC* ^3^		Antibiotic inactivation		
*fosA* ^3^	Phosphonic acid		thiol transferase	chromosome
*nfsA* ^3^	Nitrofuran	Target modification	nitroreductase	
*nfsB* ^3^				
*mexA* ^3^	Broad-spectrum	Efflux pump		chromosome
*mexB* ^3^				

^1^ ARGs identified only in isolates collected in 2022; ^2^ ARGs identified in isolates collected in 2022 and 2023; ^3^ ARGs identified only in isolates collected in 2023.

## Data Availability

All information about antibiotic resistance genes were curated from The Comprehensive Antibiotic Resistance Database (https://card.mcmaster.ca, accessed on 23 October 2024).

## Supplementary Materials

The following supporting information can be downloaded at: https://www.mdpi.com/article/10.3390/biom15010001/s1, Table S1: PCR primers used and annealing temperatures for ARG and integrase gene amplifications. Table S2. Details about the origin, and phenotypic and genotypic resistance of the *P. aeruginosa* isolates. Table S3. Antibiotics and ARGs tested in *P. aeruginosa* isolates.
