# Supplementary material for: The Difference a Year Can Make: How Antibiotic Resistance Mechanisms in Pseudomonas aeruginosa Have Changed in Northwestern Transylvania
Source: Biomolecules. 2024 Dec 24;15(1):1. doi: 10.3390/biom15010001 (PMC11762482; doi:10.3390/biom15010001)
Supplement: Supplementary file 1 [file biomolecules-15-00001-s001.zip › biomolecules-3326846-supplementary.pdf]

Table S1. PCR primers used and annealing temperatures for ARGs and integrases genes amplifications.

Antibiotic class	Gene name	Primer direction	Primer sequence (5′-3′)	Annealing temperature
Beta-lactams (penicillins)	*TEM-1*	F	GGTCGCCGCATACACTATTC	57°C
		R	ATACGGGAGGGCTTACCATC	
	SHV-1	F	GCGTTATATTCGCCTGTGTATTAT	61°C
		R	GCCTGTTATCGCTCATGGTAATG	
Beta-lactams (cephalosporines)	*PER-1*	F	AAAAACAGCAACCAGCAAGG	53°C
		R	TTACTGCCTCGACGCTACTG	
	AmpC	F	AGAAGGACCAGGCACAGATC	57°C
		R	CTCGGCATTGGGATAGTTGC	
	CTX-M	F	GTGAAACGCAAAAGCAGCTG	55°C
		R	CCGGTCGTATTGCCTTTGAG	
Beta-lactams (carbapenems)	*KPC*	F	CGTCTAGTTCTGCTGTCTT	53°C
		R	CTTGTCATCCTTGTTAGGCG	
	VIM-1	F	GATGGTGTTTGGTCGCATATC	57°C
		R	ACGGCACAACCACCGTATAG	
	GES-4	F	TATGCATCGGAAAAATTAACCTTC	56°C
		R	AGGCGTAGTTGTATCTCTGAGGTC	
	IMP	F	ACCGCAGCAGAGTCTTTGCC	56°C
		R	ACAACCAGTTTTGCCTTACC	
	NDM	F	GGTTTGGCGATCTGGTTTTC	57°C
		R	CGGAATGGCTCATCACGATC	
	NDM-1	F	GGTTTGGCGATCTGGTTTTC	55°C
		R	CGGAATGGCTCATCACGATC	
	OXA-48	F	ATGGCAAGAAAACAAAAGTTGG	53°C
		R	TTGAGCACTTCTTTTGTGATGG	
	OXA-50	F	GAAAGGCACCTTCGTCCTCTAC	61°C
		R	CAGAAAGTGGGTCTGTTCCATC	
	PstS	F	GGCTTTCGAGCAGAAGTACG	57°C
		R	ATGTAGCCGTCCTTGACCAC	
Aminoglycosides	*aac(6′)-Im*	F	GGCTGACAGATGACCGTGTTCTTG	61°C
		R	GTAGATATTGGCATACTACTCTGC	
	aac(3′)-IIIa	F	GCATGCCTCACTTAAAGCGA	55°C
		R	ACCGTTTCTTCCAAGCATCG	
	aac(6′)-II	F	AGCGACCGACTCTTGATGAA	53°C
		R	GGCTTGTCGTGTTTGAACC	
	ant(4′)-Ia	F	GTCAAAAACTGCTAACACAAG	53°C

		R	AATAATACTGCTAACGATAAT	
	AAC(6′)-Ie-APH(2′′)-Ia	F	CCAAGAGCAATAAGGGCATA	53°C
		R	CACTATCATAACCACTACCG	
	aph(2′′)-Ib	F	CTGAACACAGCAGCGACTAC	55°C
		R	TTGTAATCGCCATGCACCAG	
	aac(3)-I	F	ACCTACTCCCAACATCAGCC	55°C
		R	TCTTCCCGTATGCCCAACTT	
	armA	F	TGGGAAGTTAAAGACGACGA	56°C
		R	CCATTCCCTTCTCCTTTCCA	
Sulfonamides	*sul1*	F	AGGCATGATCTAACCCTCGG	57°C
		R	GGCCGATGAGATCAGACGTA	
	sul2	F	GACAGTTATCAACCCGCGAC	57°C
		R	GAAACAGACAGAAGCACCGG	
	sul3	F	GTGGGCGTTGTGGAAGAAAT	55°C
		R	AAAAGAAGCCCATACCCGGA	
Tetracyclines	*tetA*	F	GCAAGCAGGACCATAATCGG	57°C
		R	GCCGATATCACAGATGGGGA	
	tetB	F	GGTTAGGGGCAAGTTTTGGG	57°C
		R	ATCCCACCACCAGCCAATAA	
	tetC	F	TGAGATCTCGGGAAAAGCGT	53°C
		R	AAAGCCGCGGTAAATAGCAA	
	tetK	F	AGGATCTGCTGCATTCCCTT	58°C
		R	AGCAAACTCATTCCAGAAGCA	
	tetL	F	TATTCAAGGGGCTGGTGCAG	57°C
		R	CGGCAGTACTTAGCTGGTGA	
	tetM	F	CCGTCTGAACTTTGCGGAAA	55°C
		R	CAACGGAAGCGGTGATACAG	
Quinolones	*qnrA*	F	AGTTTGATGGTTGCCGCTTT	53°C
		R	TCTTCATTGATCTGCACGCC	
	qnrB	F	TCGTGCGATGCTGAAAGATG	55°C
		R	CCGAATTGGTCAGATCGCAA	
	qnrS	F	TGATCTCACCTTCACCGCTT	55°C
		R	GAGTTCGGCGTGGCATAAAT	
Macrolids	*ermA*	F	GAACCAGAAAAACCCTAAAGACAC	51°C
		R	ACAGAGTCTACACTTGGCTTAGGATG	
	ermB	F	GAAAAGGTACTCAACCAAATA	61°C
		R	AGTAACGGTACTTAAATTGTTTAC	
	ermC	F	CGTAACTGCCATTGAAATAGACC	61°C

		R	GTGAGCTATTCACTTTAGGTTTAGG	
	mphC	F	GAGACTACCAAGAAGACCTGACG	56°C
		R	CATACGCCGATTCTCCTGAT	
	mefA	F	CATCGACGTATTGGGTGCTG	55°C
		R	CCGAAAGCCCCATTATTGCA	
Nitrofurans	*nfsA*	F	CTGGCGCTTGCTCTGCTATC	59°C
		R	GCCCGCGTATCATACACTGG	
	nfsB	F	ATCACCGTCTCGCTACTCAAC	59°C
		R	CGCGCCATTGATCATTGAGG	
Glycopeptides	*vanA*	F	GCTATTCAGCTGTACT	51°C
		R	CAGCGGCCATCATACGG	
	vanB	F	CGCCATATTCTCCCCGGATAG	61°C
		R	AAGCCCTCTGCATCCAAGCAC	
Phosphonic Acid	*fosA*	F	GCTGCACGCCCGCTGGAATA	65°C
		R	CGACGCCCCCTCGCTTTTGT	
Wide spectrum	*MexA*	F	ATCAACCTGCGCTACACCAAG	61°C
		R	AGGCCTTCGGTAATGATCTTGT	
	MexB	F	TTTCATTGATAGGCCCATTTTC	57°C
		R	AGGGTCTTCACTACCTCATGGA	
Integrons	*int1*	F	CAGTGGACATAAGCCTGTTC	56°C
		R	CCCGACGCATAGACTGTA	
	int2	F	TTGCGAGTATCCATAACCTG	
		R	TTACCTGCACTGGATTAAGC	
	int3	F	GCCTCCGGCAGCGACTTTCAG	
		R	ACGGATCTGCCAAACCTGACT	

Table S2. Details about the origin, phenotypic and genotypic resistance of the *P. aeruginosa* isolates.

No.	Isolate	Origin	Collection date	Phenotypic resistance	Phenotypic susceptibility	Genotypic resistance (ARGs)
1	P1_23	urine	28/04/23	amikacin, cefepime, ceftazidime, ciprofloxacin, imipenem, meropenem, piperacillin-tazobactam, piperacillin, tobramycin	colistin	*aac(3)-I, OXA-50, sul1, tetA, tetK, qnrB, fosA, nfsA*
2	P2_23	urine	31/03/23	amikacin, cefepime, ceftazidime, ciprofloxacin, imipenem, meropenem, piperacillin-tazobactam, piperacillin, tobramycin	colistin	*OXA-50, sul1*
3	P3_23	urine	23/04/23	amikacin, cefepime, ceftazidime, ciprofloxacin, imipenem, meropenem, piperacillin-tazobactam, piperacillin, tobramycin	colistin	*OXA-50, TEM-1*
4	P4_23	purulent collection	06/07/23	amikacin, cefepime, ceftazidime, ciprofloxacin, imipenem, piperacillin-tazobactam, piperacillin, tobramycin	colistin	*aac(6')-II, ant(4')-Ia, AAC(6')-Ie-APH(2'')-Ia, sul1, tetK, qnrB, ermB, ermC, mphC, fosA, nfsA, nfsB, mphC*
5	P5_23	purulent collection	24/02/23	amikacin, cefepime, ceftazidime, ciprofloxacin, imipenem, meropenem, piperacillin, tobramycin	colistin, piperacillin-tazobactam	*OXA-50, TEM-1, sul1, tetA, tetK, nfsB, mexB*
6	P6_23	urine	08/03/23	amikacin, cefepime, ceftazidime, ciprofloxacin, imipenem, meropenem, piperacillin-tazobactam, piperacillin, tobramycin	colistin	*OXA-50, TEM-1, sul1, tetA, tetK, fosA, nfsA, mexA*
7	P7_23	urine	08/03/23	amikacin, cefepime, ceftazidime, ciprofloxacin, imipenem, meropenem, piperacillin-tazobactam, piperacillin, tobramycin	colistin	*OXA-50, VIM-1, TEM-1, sul1, mexB*
8	P8_23	purulent collection	02/04/23	amikacin, cefepime, ceftazidime, ciprofloxacin, imipenem, meropenem, piperacillin-tazobactam, piperacillin, tobramycin	colistin	*OXA-50, sul1, qnrB*
9	P9_23	urine	09/03/23	amikacin, cefepime, ceftazidime, ciprofloxacin, imipenem, meropenem, piperacillin-tazobactam, piperacillin, tobramycin	colistin	*aac(6')-Im, OXA-50, TEM-1, sul1, tetA, qnrB, nfsA*
10	P10_23	urine	02/02/23	amikacin, cefepime, ceftazidime, ciprofloxacin, colistin, imipenem, meropenem, piperacillin-tazobactam, piperacillin, tobramycin	-	*aac(6')-II, ant(4')-Ia, AAC(6')-Ie-APH(2'')-Ia, OXA-50, TEM-1, sul1, tetK, ermB, ermC, fosA, nfsA, nfsB, mexA, mexB*
11	P11_23	urine	27/04/23	amikacin, cefepime, ceftazidime, ciprofloxacin, imipenem, meropenem, piperacillin-tazobactam, piperacillin, tobramycin	colistin	*aph(2'')-Ib, aac(6')-Im, OXA-50, VIM-1, ampC, sul1, sul2, tetK, qnrB, ermB, mphC, fosA, nfsA, nfsB, mexA, mexB*
12	P12_23	urine	09/02/23	amikacin, cefepime, ceftazidime, ciprofloxacin, imipenem,	colistin, tobramycin	*aph(2'')-Ib, aac(3)-I, OXA-50, VIM-1, ampC, sul1, sul2, tetC, tetK, qnrB, ermB, mphC, fosA, nfsA, nfsB, mexA*

				meropenem, piperacillin-tazobactam, piperacillin		
13	P1_22	tracheal aspirate	05/04/22	piperacillin	amikacin, cefepime, ceftazidime, ciprofloxacin, colistin, imipenem, meropenem, piperacillin-tazobactam, tobramycin	*aph(2'')-Ib, amp, sul1*
14	P2_22	tracheal aspirate	22/09/22	-	amikacin, cefepime, ceftazidime, ciprofloxacin, colistin, imipenem, meropenem, piperacillin-tazobactam, piperacillin, tobramycin	*OXA-50, amp, ermA*
15	P3_22	urine	27/07/22	amikacin, cefepime, ceftazidime, ciprofloxacin, imipenem, meropenem, piperacillin, tobramycin	colistin, piperacillin-tazobactam	*aph(2'')-Ib, ermA*
16	P4_22	urine	13/05/22	amikacin, cefepime, ceftazidime, ciprofloxacin, imipenem, meropenem, piperacillin, tobramycin	colistin, piperacillin-tazobactam	*amp*
17	P5_22	blood	12/08/22	amikacin, cefepime, ceftazidime, ciprofloxacin, imipenem, meropenem, piperacillin	colistin, piperacillin-tazobactam, tobramycin	-
18	P6_22	tracheal aspirate	27/09/22	-	amikacin, cefepime, ceftazidime, ciprofloxacin, colistin, imipenem, meropenem, piperacillin-tazobactam, piperacillin, tobramycin	*ermA*
19	P7_22	plague secretion	01/07/22	-	amikacin, cefepime, ceftazidime, ciprofloxacin, colistin, imipenem, meropenem, piperacillin-tazobactam, piperacillin, tobramycin	-
20	P8_22	blood	01/08/22	-	amikacin, cefepime, ceftazidime, ciprofloxacin, colistin, imipenem, meropenem, piperacillin-tazobactam, piperacillin, tobramycin	-
21	P9_22	urine	01/12/22	-	amikacin, cefepime, ceftazidime, ciprofloxacin, colistin, imipenem, meropenem, piperacillin-tazobactam, piperacillin, tobramycin	*amp, fosA*
22	P10_22	punction liquid	14/01/22	-	amikacin, cefepime, ceftazidime, ciprofloxacin, colistin, imipenem, meropenem, piperacillin-tazobactam, piperacillin, tobramycin	*OXA-50, amp*
23	P11_22	pleural liquid	20/01/22	-	amikacin, cefepime, ceftazidime, ciprofloxacin, colistin, imipenem, meropenem, piperacillin-	*OXA-50, tetA*

					tazobactam, piperacillin, tobramycin	
24	P12_22	plague secretion	29/01/22	-	amikacin, cefepime, ceftazidime, ciprofloxacin, colistin, imipenem, meropenem, piperacillin-tazobactam, piperacillin, tobramycin	*OXA-50, ermA*
25	P13_22	tracheal aspirate	02/03/22	-	amikacin, cefepime, ceftazidime, ciprofloxacin, colistin, imipenem, meropenem, piperacillin-tazobactam, piperacillin, tobramycin	*aph(2'')-Ib, OXA-50, amp, sul1*
26	P14_22	plague secretion	17/2/2022	amikacin, ciprofloxacin, imipenem, tobramycin	cefepime, ceftazidime, colistin, meropenem, piperacillin-tazobactam, piperacillin,	*OXA-50, ampC*
27	P15_22	blood	25/2/2022	-	amikacin, cefepime, ceftazidime, ciprofloxacin, colistin, imipenem, meropenem, piperacillin-tazobactam, piperacillin, tobramycin	*OXA-50, ampC, amp, tetA, ermB*
28	P16_22	tracheal aspirate	03/01/22	amikacin, cefepime, ceftazidime, ciprofloxacin, imipenem, meropenem, piperacillin-tazobactam, piperacillin, tobramycin	colistin	*aph(2'')-Ib, OXA-50, ampC, ermB*
29	P17_22	plague secretion	03/09/22	amikacin, cefepime, ceftazidime, ciprofloxacin, imipenem, meropenem, piperacillin-tazobactam, piperacillin, tobramycin	colistin	*aph(2'')-Ib, OXA-50, ampC, amp, sul1*
30	P18_22	tracheal aspirate	17/03/22	amikacin, cefepime, ceftazidime, ciprofloxacin, imipenem, meropenem, piperacillin-tazobactam, piperacillin, tobramycin	colistin	*OXA-50, ampC, amp*
31	P19_22	plague secretion	22/03/22	amikacin, cefepime, ceftazidime, ciprofloxacin, imipenem, meropenem, piperacillin-tazobactam, piperacillin, tobramycin	colistin	*OXA-50, ampC, amp, tetA, ermA*
32	P20_22	urine	25/03/22	amikacin, cefepime, ceftazidime, ciprofloxacin, imipenem, meropenem, piperacillin-tazobactam, piperacillin, tobramycin	colistin	*OXA-50, ermA*
33	P21_22	plague secretion	04/07/22	amikacin, cefepime, ceftazidime, ciprofloxacin, colistin, piperacillin-tazobactam, piperacillin, tobramycin	imipenem, meropenem	*aph(2'')-Ib, OXA-50, sul1*
34	P22_22	blood	19/04/22	cefepime, ceftazidime, ciprofloxacin, colistin, piperacillin-tazobactam, piperacillin	amikacin, imipenem, meropenem, tobramycin	*OXA-50, amp, tetA*

Table S3. Antibiotics and ARGs tested in *P. aeruginosa* isolates.

No.	Antibiotic	Antibiotic class	ARG
1	amikacin	aminoglycoside	*ant(4′)-Ia, aac(6′)-Im, armA*
2	tobramycin	aminoglycoside	*aac(6′)-Im, ant(4′)-Ia, aac(3)-IIIa, aac(6′)-II*
3	piperacillin	beta-lactam	*ampC*
4	cefepime	beta-lactam	*ampC*
5	ceftazidime	beta-lactam	*mexA, mexB, KPC, CTX-M*
6	imipenem	beta-lactam	*ampC, NDM-1*
7	meropenem	beta-lactam	*MexA, MexB, NDM-1*
8	ciprofloxacin	fluorochinolone	*qnrA, qnrB, qnrS*
9	colistin	polimixin	*mexA, mexB*
10	piperacillin-tazobactam	See piperacillin	
